# Novel Compound Heterozygous CEP152 Variants Associated with Atypical Seckel Syndrome 5 in a Chinese Child: Functional Characterization of a Novel Splice-Site Variant and Expansion of the Genotype–Phenotype Spectrum

**DOI:** 10.3390/genes17101249

**Published:** 2026-10-10

**Authors:** Mu Du, Pengchao Wang, Ziqin Liu, Bingyan Cao

**Affiliations:** 1 Clinic of Endocrinology, Capital Center For Children’s Health, Capital Medical University, Capital Institute of Pediatrics, No. 2 Yabao Road, Chaoyang District, Beijing 100020, China; dumu19850718@163.com; 2 Chigene (Beijing) Translational Medical Research Center Co., Ltd., No. 88, Kechuang Sixth Street, Beijing Economic-Technological Development Area, Beijing 100176, China; wangpengchaobj@163.com

**Keywords:** CEP152 gene, Seckel syndrome (SCKL5), microcephaly, whole-exome sequencing, minigene assay

## Abstract

**Background**: Seckel syndrome (SCKL) is a rare autosomal-recessive inherited disorder characterized by intrauterine and postnatal growth restriction, microcephaly, “bird-like” facial dysmorphisms, and mild-to-moderate intellectual disability (ID). CEP152 is one of the known causative genes, and pathogenic variants in CEP152 are responsible for SCKL5. To date, 18 CEP152 variants associated with SCKL5 have been reported in the peer-reviewed literature. According to the Human Gene Mutation Database (HGMD, version 2026.4), 51 CEP152 variants have been cataloged, including 28 disease-causing mutations (DMs) and 23 putative disease-causing mutations (DMs?). **Methods**: We conducted a retrospective analysis of a Chinese boy with suspected SCKL5 who was seen at our hospital in 2024, based on a review of his detailed clinical, genetic, and family history. Whole-exome sequencing (WES) followed by Sanger sequencing identified two novel CEP152 variants. A minigene splicing assay was performed to confirm the functional effect of the splice-site variant. We further analyzed the clinical phenotype of patients with SCKL5 carrying CEP152 variations based on the published literature. **Results**: Two novel compound heterozygous CEP152 (NM_014985.4) variants were identified: the paternal c.3661delA (p.(I1221Lfs*20)) and the maternal c.1577+3A>G. An in vitro minigene assay showed that c.1577+3A>G leads to aberrant splicing, including intron 12 retention (77 bp) and exon 12 skipping. Both splicing alterations introduce premature termination codons and are predicted to produce truncated protein. **Conclusions**: We discovered two novel CEP152 variants, c.3661delA and c.1577+3A>G, in a Chinese patient with SCKL5. Moreover, retention of intron 12 or exon 12 skipping due to the c.1577+3A>G variant was revealed by the minigene assay. These findings expand the variant and phenotypic spectrum of CEP152 and provide valuable insights for genetic counseling.

## 1. Introduction

Seckel syndrome (SCKL) is a rare autosomal recessive neurodevelopmental disorder, also known as “bird-headed dwarfism” or microcephalic primordial dwarfism. Its estimated incidence is less than 1 per 10,000 individuals [1]. It was first described by Seckel in 1960, characterized by severe prenatal and postnatal growth restriction; severe microcephaly; ID; and typical “bird-like” facial dysmorphisms including a sloping forehead, micrognathia, a beaked nose, and a high nasal bridge [2,3,4]. Additional reported features include low birth weight [5], central nervous system vasculopathy [6], cardiovascular anomalies [7,8], and myelodysplasia [9]. To date, nine genetic subtypes of SCKL have been identified, caused by variants in ATR, RBBP8, CENPJ, CEP152 (OMIM: 613529), CEP63, NIN, DNA2, TRAIP, and NSMCE2. Homozygous or compound heterozygous loss-of-function pathogenic variants in CEP152 lead to autosomal recessive SCKL5 (OMIM: 613823) [3].

CEP152, located at 15q21.1, contains 26 exons in total and encodes a 1654-amino-acid centrosomal protein (~152 kDa) essential for centrosome assembly and duplication and thus required for cell division in animal cells [3,10,11,12]. It has also been confirmed to play a key role in maintaining genome integrity and the DNA damage response [13,14]. Since Ersan Kalay first reported the CEP152 variant in 2011 [15], 18 variations have been documented in the peer-reviewed literature in patients with SCKL5, including six frameshift variants, four nonsense variants, three missense variants, one *CEP152* whole-gene deletion, and four splice-site variants. According to the Human Gene Mutation Database (HGMD, version 2026.4), a total of 51 CEP152 variants are catalogued, including 28 disease-causing mutations (DMs) and 23 putative disease-causing mutations (DMs?). The ClinVar database contains 143 CEP152 pathogenic (P) or likely pathogenic (LP) variant entries; only a small subset of these entries are associated with SCKL5, and these overlapping variants are already included in the HGMD dataset. Most P/LP entries are submitted by clinical diagnostic laboratories without detailed patient phenotype information (“not provided”). Therefore, no additional SCKL5-associated variants were retrieved from ClinVar in the present study.

In this study, we report a Chinese boy with SCKL5 who was born to healthy, nonconsanguineous parents. He harbors two novel CEP152 variants, which together constitute a compound heterozygous genotype. Using a minigene assay, we further validated the functional effect of the splice-site variant and compared our findings with previously reported SCKL5 cases to more precisely define the genotype–phenotype spectrum. Our finding expands the genotypic and phenotypic spectrum associated with CEP152.

## 2. Materials and Methods

### 2.1. Patients

Written informed consent was obtained from the proband’s parents (as legal guardians) for the publication of this study, including all accompanying clinical data, genetic information, and potentially identifiable images. Ethical approval was granted by the Ethics Committee of the Capital Institute of Pediatrics (Approval No. SHERLLM2025056). The proband was a boy aged 2 years and 9 months who presented to our hospital with short stature. Prenatal genetic evaluations, including amniocentesis-derived karyotype and copy number variation sequencing (CNV-seq), were normal. Postnatally, based on his clinical presentation, the patient underwent comprehensive physical examination, laboratory evaluations (including thyroid function, IGF-1, and IGFBP-3), and imaging studies (such as pituitary MRI, skeletal radiographs, and an anteroposterior left wrist radiograph for bone age assessment).

### 2.2. Whole-Exome Sequencing and Data Analysis

According to the manufacturer’s protocol, genomic DNA was extracted from peripheral blood samples using a blood genome extraction kit (Tiangene, Beijing, China). Whole-exome sequencing (WES) was performed using DNBSEQ-T7 (MGI Tech Co., Ltd., Shenzhen, China) with the xGen Exome Research Panel v2.0 (IDT, Coralville, IA, USA) at Chigene (Beijing) Translational Medical Research Center Co., Ltd. Sequencing reads were processed, and fastq files were generated according to the manufacturer’s instructions. Reads were aligned to GRCh37/hg19 using BWA, and SNVs/Indels were called using GATK (4.1.8.1) software. The proband’s WES achieved a mean depth of 208×, with 99.2% of the exome covered at 20×. For CEP152, the mean sequencing depth was 223×, with 100% of the target region covered at >10×. Variant pathogenicity was assessed using bioinformatics tools such as Provean, SIFT, Polyphen2 (HDIV and HVAR), M-CAP, REVEL, CADD, SpliceAI, and MaxEntScan and annotated against dbSNP, the 1000 Genome Project, ExAC, ESP, gnomAD, and an in-house database from Chigene Co., Ltd. (WES data from 230,000 patients with diverse disorders and 230,000 controls). Finally, the variants were classified according to the American College of Medical Genetics and Genomics/Association for Molecular Pathology (ACMG/AMP) guidelines [16]. Sanger sequencing was used to further validate the candidate variants in the proband and his parents, and Primer 5.0 software was used to design the CEP152 primers: c.3661delA forward primer (5′-GATTCTCACATCACTTCCAAAAG-3′) and reverse primer (5′-CTGTATAGTGGAGATTGTTCTAGATG-3′); c.1577+3A>G forward primer (5′-TGAAAACCTGATATATGCCGACCT-3′) and reverse primer (5′-CGCTCTCACCTTAAAACAGATACC-3′). Amplicons were run on an ABI 3730XL DNA Analyzer (Applied Biosystems, Carlsbad, CA, USA). Both variants were submitted to the ClinVar database (https://www.ncbi.nlm.nih.gov/clinvar/, accessed on 7 October 2026) (accession numbers: SCV006324105 and SCV006324106). Gene structure and domain model diagrams were created using IBS 2.0 (https://ibs.renlab.org/#/server, accessed on 18 September 2025) [17].

### 2.3. In Vitro Minigene Splicing Assay

To investigate the effect of c.1577+3A>G on pre-mRNA splicing, we performed an in vitro minigene splicing assay. We generated a minigene construct in the pcDNA3.1 vector, incorporating CEP152 Exon 11 (92 bp)-Intron 11 (779 bp)-Exon 12 (164 bp)-partial Intron 12 (1177 bp)-Exon 13 (205 bp) (GRch37:chr15:48772487-48772691,48780019-48782230). This construct preserves the core cis-acting elements required for recognizing the c.1577+3A>G 5′ donor splice site. After confirmation by PCR and Sanger sequencing, the pcDNA3.1-CEP152-wild-type (wt) and mutant (mut) plasmids were transfected into HeLa and 293T cells in accordance with the manufacturer’s protocol. Notably, the wild-type minigene construct produced correctly spliced transcripts in cellular assays, confirming that this vector provides a functional splicing-competent context that reproduces the physiological splicing pattern of this genomic region. Total RNA was extracted 48 h later with TRIzol reagent (Invitrogen, Carlsbad, CA, USA), reverse-transcribed, and amplified by RT-PCR using the vector-specific primers pcDNA3.1-F (5′-GCTTGGTACCATGGGGTCAGTACAAGAGGTGGCT-3′) and pcDNA3.1-R (5′-TAGACTCGAGCTCAACCTCAATGCTTTTTT-3′). Finally, PCR products were separated using agarose gel electrophoresis. RT-PCR and agarose gel electrophoresis for the minigene assay were conducted without biological replicates. This gel-based assay is semi-qualitative and designed to detect the occurrence of aberrant splicing. Individual PCR bands were purified and Sanger-sequenced to verify the identity of splicing products so as to define the splicing pattern of Exons 11-13 and identify any aberrant splicing events.

## 3. Results

### 3.1. Clinical Presentation

**Figure 1:**
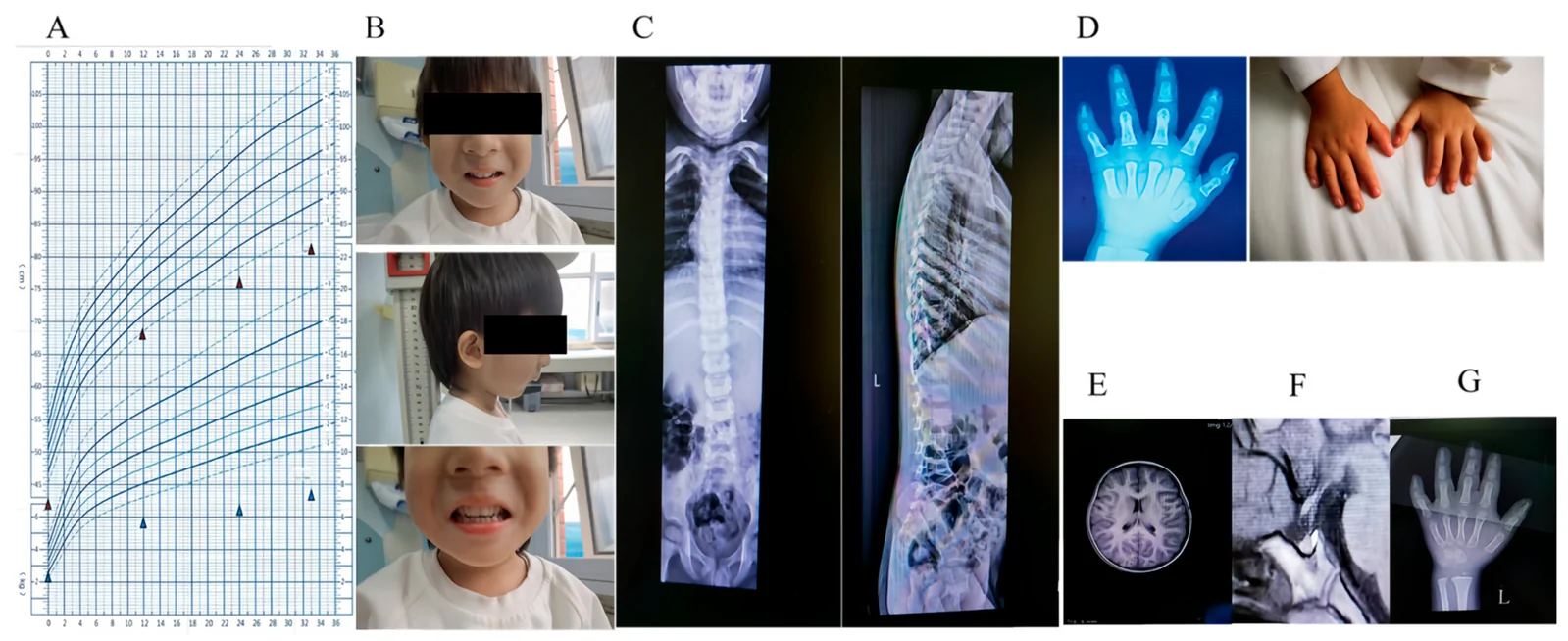
Clinical and radiographic findings of the proband. (**A**) Growth chart from birth to 2 years and 9 months of age. Longitudinal height and weight measurements demonstrate severe pre- and postnatal growth restriction, persistently falling below the −3 SD reference curves for Chinese children. Red triangles indicate height for age and blue triangles indicate weight for age. (**B**) Facial features showing a sloping forehead, a prominent nasal bridge, and low-set ears. The eyes and adjacent regions are obscured to protect the pediatric patient’s privacy in accordance with ethical guidelines. An intraoral photograph of the high-arched palate could not be obtained due to the young patient’s lack of cooperation; however, this feature was confirmed during physical examination. (**C**) Spinal radiographs. (**D**) Hand radiograph and photograph, showing bilateral clinodactyly of the fifth finger. The radiograph also indicates a bone age of 2 years and 6 months. (**E**) Axial brain MRI. (**F**) Sagittal pituitary MRI. (**G**) Bone age assessment at the chronological age of 3 years and 11 months, quantified as 3 years and 7 months using the Tanner–Whitehouse 3 (TW3) method.

The proband was the firstborn child of non-consanguineous Chinese parents. He was delivered at 37 + 2 weeks of gestation via an uncomplicated delivery. At birth, his anthropometric measurements were markedly abnormal: he had a body length of 42 cm (−4.67 SD), a weight of 2050 g (−3.26 SD), and a head circumference (HC) of 30.5 cm (−3.3 SD). Retrospective data confirmed that his growth remained severely restricted during early childhood. By 1 year of age, his height was 68 cm (−3.15 SD) and his weight was 5.9 kg (−3.5 SD); at 2 years of age, these measurements were 76 cm (−3.68 SD) and 6.2 kg (−4.88 SD), respectively (Figure 1A).

At 2 years and 9 months of age, the patient presented to our endocrinology clinic with severe failure to thrive, prompting the genetic investigations that ultimately led to his diagnosis. At this initial assessment, his height was 81 cm (−3.71 SD), his weight was 7.5 kg (−4.7 SD), and his HC was 37.5 cm (−8.92 SD) (Figure 1A). Physical examination revealed typical “bird-like” craniofacial features characteristic of Seckel syndrome, including microcephaly, a triangular face, micrognathia, small and down-slanting palpebral fissures, a prominent nasal bridge, a sloping forehead, low-set ears, and a high-arched palate (Figure 1B). He also exhibited small hands and bilateral clinodactyly of the fifth fingers (Figure 1D). Despite these profound physical findings, his motor and language development were age-appropriate.

Concurrent clinical and functional evaluations revealed no significant abnormalities on brain and pituitary magnetic resonance imaging (MRI) (Figure 1E,F). Anteroposterior and lateral spinal radiographs demonstrated mild scoliosis (Figure 1C). Baseline laboratory tests showed normal thyroid function (FT4, 13.5 pmol/L; FT3, 5.94 pmol/L; TSH, 3.45 mIU/L) and a normal insulin-like growth factor-binding protein 3 (IGFBP-3) level (2478 ng/mL). His initial insulin-like growth factor 1 (IGF-1) level was 47.2 ng/mL. An initial skeletal survey indicated a bone age of 2 years and 6 months, which was slightly delayed but largely commensurate with his chronological age (Figure 1D). A growth hormone (GH) stimulation test was deferred due to the patient’s young age.

At the latest follow-up (3 years and 11 months of age), his anthropometric parameters remained severely restricted, with a height of 86.4 cm (−4.38 SD), weight of 10 kg (−4.78 SD), and HC of 40.5 cm (−7.54 SD) (Figure 1A). Endocrine reassessment yielded an IGF-1 concentration of 185.67 ng/mL, which is well within the age-appropriate reference range (49–311 ng/mL). Given this normal IGF-1 level, a GH stimulation test was not pursued. Repeat radiography demonstrated a bone age of 3 years and 7 months, remaining essentially concordant with his chronological age (Figure 1G).

We systematically reviewed previously reported SCKL5 cases with complete clinical and molecular data. Literature databases, including PubMed, CNKI, Wanfang, VIP, and Chinese Medical Journal Full-text Database (CMAJD) Chinese Medical Journal, were searched. HGMD and ClinVar databases were additionally queried for variant information. The search terms used were “SCKL”, “SCKL5”, “Seckel syndrome”, and “CEP152”. The literature search cutoff was July 2026. Two investigators independently screened the titles, abstracts, and full-text articles. Case reports/series were included if they had both confirmed CEP152 pathogenic variants and adequate individual clinical phenotype data. Exclusion criteria included reviews, animal studies, cases lacking detailed clinical or molecular information, and duplicate publications. Thirteen patients (10 males and 3 females) were included in this summary analysis (Table 1). The ages of the included cases ranged from the prenatal period (31 weeks of gestation) to 20 years. Full individual-level clinical details are listed in Table 1.

All evaluated patients exhibited severe and persistent pre- and postnatal growth retardation. Birth height standard deviation scores (SDS) ranged from −2.3 to −7.0, with growth failure progressing throughout the preschool period and persisting into adulthood. Similarly, severe microcephaly was a universal finding that progressively exacerbated from infancy to mid-childhood. For instance, the proband’s head circumference SDS worsened from −3.33 at birth to −7.54 at 3 years and 11 months, mirroring the progressive trajectory observed in other reported cases.

Typical “bird-like” facial dysmorphism, including a sloping forehead, micrognathia, and a beaked nose, was universally present. As detailed in Table 1, patients frequently exhibited additional facial and dental abnormalities, such as a high-arched palate, low or malformed ears, down-slanting palpebral fissures, and selective tooth agenesis. Skeletal abnormalities were also highly prevalent, most notably fifth-finger clinodactyly, missing ribs (11 pairs), and delayed bone age. Genitourinary anomalies, including cryptorchidism, clitoromegaly, and hypospadias, were observed in more than half of the cohort. Neurologically, ID or developmental delay (DD), along with simplified gyri on neuroimaging, was highly prevalent. Notably, our proband is the only patient in this cohort currently exhibiting normal neurodevelopment with a normal Developmental Quotient (DQ) on the Gesell Developmental Schedules (GDS) at 2 years and 9 months. In conclusion, these findings highlight that affected individuals present with a broad spectrum of multisystem abnormalities, making careful and comprehensive clinical evaluation crucial for their diagnosis and management.

### 3.2. Identification of Variants in CEP152

**Figure 2:**
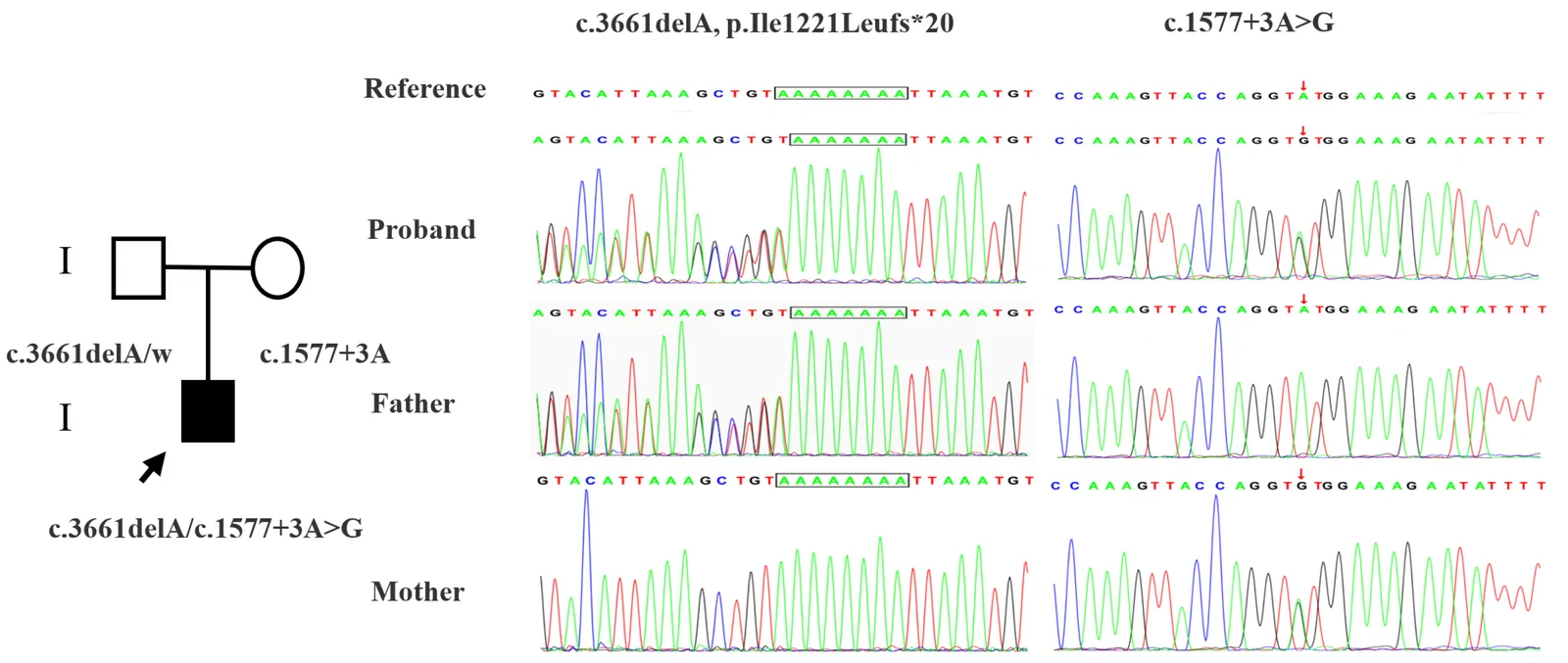
Pedigree of the family and Sanger sequencing validation for variants in CEP152. NM_014985.4. wt, wild-type allele. Red arrow: indicates the variant position.

**Figure 3:**
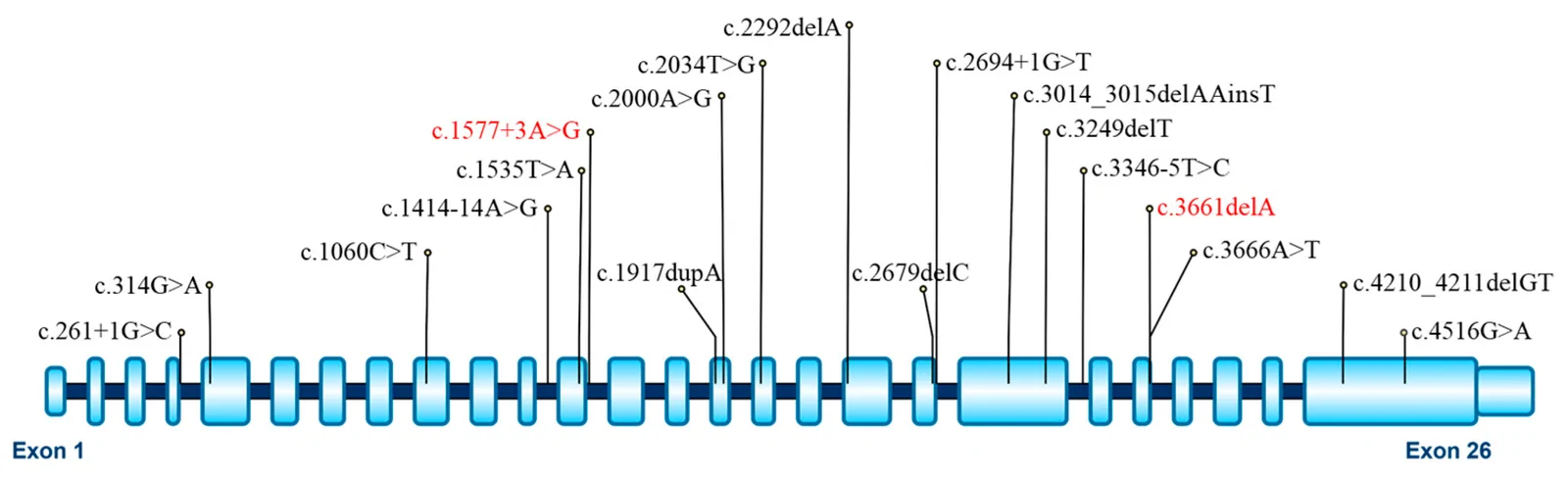
Schematic diagram of the CEP152 gene structure, showing the variants of this study (red) and other previous studies (black).

WES followed by Sanger sequencing identified two novel compound heterozygous variants of CEP152 in the patient: chr15:49036450delT(GRCh37), c.3661delA, p.I1221Lfs*20 and chr15:49073390T>C(GRCh37), c.1577+3A>G (Figure 2 and Figure 3). No additional pathogenic or likely pathogenic variants that could explain the proband’s phenotype were identified, making a second genetic diagnosis unlikely. We note that standard WES cannot reliably detect deep-intronic variants and certain copy number alterations; therefore, they cannot be formally excluded.

The c.3661delA variant is located in exon 23 of CEP152 (NM_014985.4). This frameshift variant generates a premature termination codon at p.I1221Lfs*20. Bioinformatically, the premature stop codon lies >50 nucleotides upstream of the final exon–exon junction, predicting that this mutant transcript may undergo nonsense-mediated mRNA decay (NMD). However, this NMD prediction has not been experimentally validated. Both variants have not been reported in public population databases (dbSNP, 1000 Genome Project, ExAC, ESP, gnomAD). The c.1577+3A>G variant was predicted to affect splicing using MaxEntScan (9.99->6.34), dbscSNV (0.9574|0.6780), and SpliceAI(0.52) software. According to the ACMG/AMP guidelines [16], c.3661delA was classified as “likely pathogenic” (PVS1, PM2_supporting), while c.1577+3A>G was preliminarily classified as “uncertain significance” (PM2_Supporting, PP3, PM3) before minigene functional testing.

### 3.3. Minigene Splicing Assay

**Figure 4:**
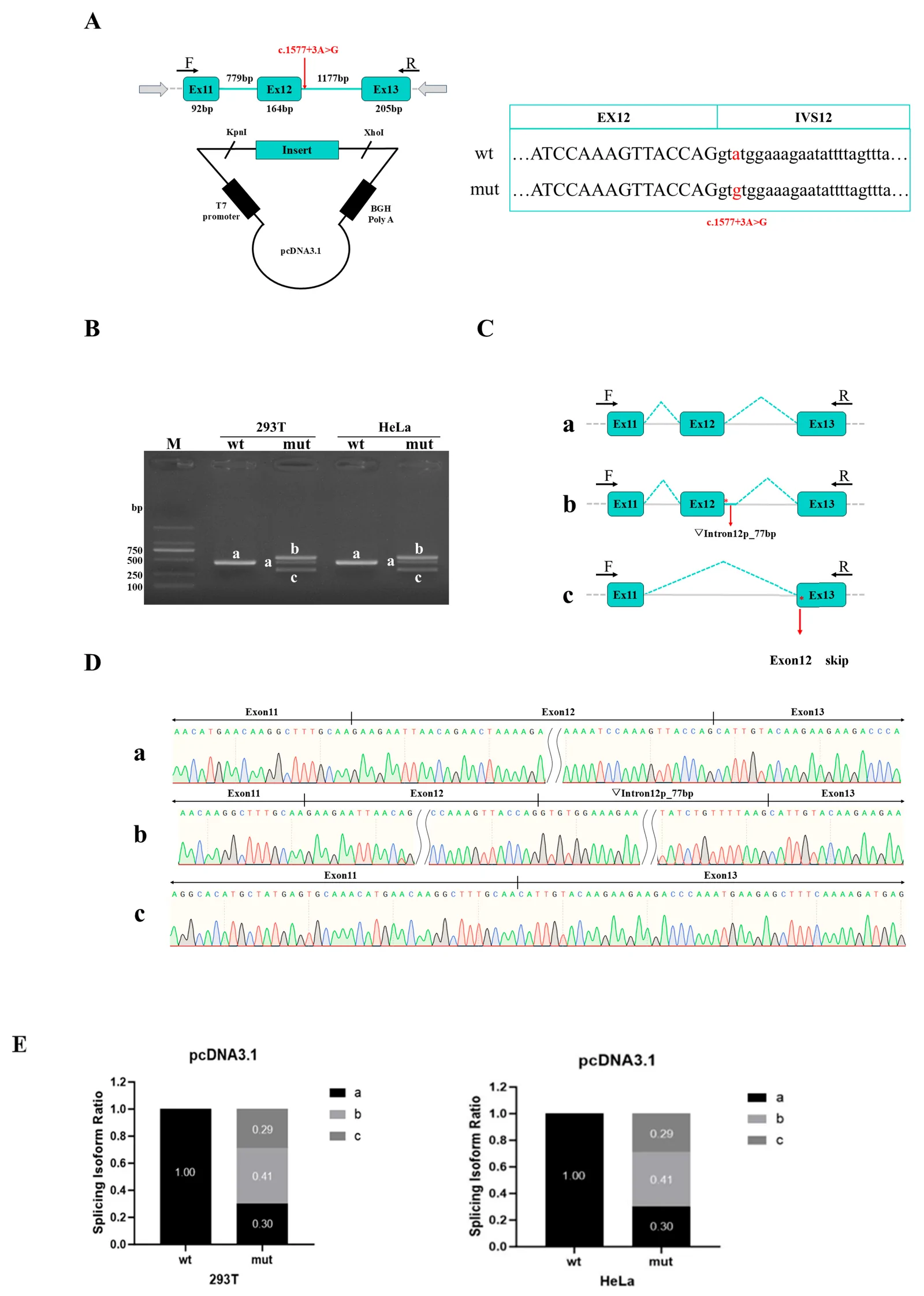
Minigene assay results for the c.1577+3A>G variant. (**A**) Schematic diagram of the pcDNA3.1-CEP152 minigene construct. F = forward primer, R = reverse primer. Red arrows mark the c.1577+3A>G variant site. (**B**) RT-PCR products from 293T and HeLa cells transfected with wt or mut pcDNA3.1-CEP152 vectors were separated by 2% agarose gel electrophoresis. Three distinct bands were detected in the mutant group, indicating the presence of multiple splicing isoforms. (**C**) Schematic of the three transcript isoforms, showing the predicted fragment sizes. * marks the position of the predicted premature termination codon (PTC) in each aberrant isoform. The retained 77 bp intronic sequence in isoform b and exon 12 deletion in isoform c introduce PTCs; these transcripts are predicted to undergo nonsense-mediated mRNA decay (NMD) and produce truncated proteins. (**D**) Sanger sequencing chromatograms confirming the splice junctions and verifying the sequence of bands a, b, and c. (**E**) Stacked bar charts showing densitometric quantification of splicing isoforms in 293T (**left**) and HeLa (**right**) cells. All proportions were normalized to total transcript abundance within each cell line.

The pcDNA3.1-CEP152-wt/mut minigene was constructed as shown in Figure 4A.

RT-PCR revealed a single expected band around 485 bp in the lane of the wild-type cDNA, while two additional smaller aberrant bands were observed in the lane of the mutant cDNA, suggesting that the c.1577+3A>G variant disrupts normal pre-mRNA splicing (Figure 4B). The full uncropped original gel image underlying Figure 4B is provided in Supplementary Figure S1. Sanger sequencing further confirmed that these aberrant isoforms corresponded to intron 12 retention (77 bp) and exon 12 skipping. Intron 12 retention inserts a 77 bp intronic sequence into mature mRNA, causing a reading-frame shift that generates the premature termination codon (PTC) p.Ser526Argfs*26, theoretically yielding a 550-amino-acid truncated protein. Exon 12 skipping removes the entire 164 bp exon 12 sequence, also leading to frame disruption and producing the PTC p.Glu472Hisfs*9, which is predicted to result in a 479-amino-acid truncated protein (Figure 4C,D). Both PTCs are situated more than 50 nucleotides upstream of the final exon–exon junction, suggesting that these mutant transcripts may be subjected to nonsense-mediated mRNA decay (NMD), but this prediction requires experimental validation. We further performed densitometric quantification of RT-PCR products extracted from minigene-transfected 293T and HeLa cells. The relative abundance of each splicing isoform was normalized to the total transcript signal per cell line, and quantitative proportions were visualized as stacked bar charts (Figure 4E, left: 293T; right: HeLa). Consistent splicing defects were observed in both cell lines: ~30% of transcripts maintained canonical splicing, while ~70% corresponded to aberrant isoforms. These quantitative data demonstrate the robust splice-disrupting activity of c.1577+3A>G in this in vitro minigene system. Raw densitometry data underlying Figure 4E from this single experiment are provided in Table S2. When incorporating minigene-derived RNA functional evidence, we followed the ACMG/AMP 2015 guidelines and the 2023 consensus recommendations from the ClinGen Splicing-Variant Interpretation (SVI) Splicing Subgroup [22]. This guideline does not establish universal percentage cutoffs for RNA splicing assay outcomes; nevertheless, its decision-tree workflow permits assignment of baseline PVS1_Strong (RNA) for out-of-frame variants that generate premature termination codons under near-complete splicing-disruption scenarios. Although approximately 70% of transcripts underwent aberrant splicing in our assay, partial canonical transcripts were still detectable. Given the lack of patient-derived endogenous transcript data, the PVS1 evidence strength was therefore downgraded by one level to PVS1_Moderate (RNA). Collectively, the criteria PM2_Supporting, PM3, and the downgraded PVS1_Moderate (RNA) were applied for c.1577+3A>G. The variant retains its final formal classification as a variant of uncertain significance. The criterion-by-criterion variant assessment performed according to the 2015 ACMG/AMP guidelines with implementation of ClinGen SVI recommendations for RNA-related functional evidence is provided in Supplementary Table S1.

## 4. Discussion

Seckel syndrome (SCKL) is a rare genetic disorder characterized by severe growth retardation and microcephaly. To date, nine subtypes of SCKL, caused by different genes, have been reported. Pathogenic variants in CEP152 are primarily associated with SCKL5 and primary microcephaly 9 (MCPH9, OMIM:614852), but have also been linked to autism spectrum disorder (ASD), epileptic encephalopathy, ID, corpus callosum hypoplasia, neuromuscular disease, and atrioventricular septal defect (AVSD) [7,17,23,24,25,26]. To date, 51 CEP152 variants have been cataloged in the Human Gene Mutation Database (HGMD), with 18 confirmed to cause SCKL5 (Figure 3). A review of 11 well-described cases (Table 1) showed consistent core features of severe prenatal-onset growth retardation and microcephaly that progressively worsen after birth (Table 1). Microcephaly, a hallmark of impaired brain development, is strongly associated with profound ID and abnormal neuroimaging features such as simplified gyral patterns. In addition, typical facial dysmorphisms, including a sloping forehead, micrognathia, a beaked nose, and a prominent nasal bridge, were observed in all patients, while additional features, such as low-set or malformed ears, down-slanting palpebral fissures, blepharophimosis, selective tooth agenesis/enamel hypoplasia, and a high-arched palate, displayed variable expressivity. Neurological involvement, often manifesting as ID or delayed speech and motor development, was frequent. Simplified gyri were noted on neuroimaging in some patients. Genital anomalies including cryptorchidism, clitoromegaly, and hypospadias were also documented in some patients.

Notably, compared with previously reported cases, our proband exhibited several atypical features: the absence of strabismus, a cleft palate, and enamel hypoplasia, alongside the presence of scoliosis. Additionally, his intellectual, language, and motor development were age-appropriate, yielding a normal DQ on the Gesell evaluation, and his structural brain MRI was unremarkable. Longitudinal relative measurements of his HC revealed a distinct trajectory. At birth, the restriction in HC was less severe than his profound linear growth failure. Postnatal serial measurements, however, demonstrated a progressive restriction in head growth relative to his body length. This dynamic indicates that his microcephaly is not merely a proportional consequence of generalized somatic growth delay, but rather reflects the primary, intrinsic impairment of brain growth typical of SCKL. Consistent with our observations, a small subset of patients with SCKL may exhibit HC impairment that initially lags behind height deficits during early childhood. Delayed bone age is a classic hallmark of Seckel syndrome; therefore, the concordant skeletal maturation observed in this patient represents an atypical phenotype. His repeatedly normal IGF-1 levels render growth hormone deficiency (GHD) an unlikely cause of the severe growth restriction. We postulate that his profound growth impairment predominantly arises from intrinsic defects in cellular proliferation—secondary to pathogenic CEP152 variants disrupting centrosomal function—rather than from secondary endocrine dysfunctions like GHD. This inherent cellular defect severely restricts longitudinal bone growth but does not necessarily decelerate skeletal maturation, thereby explaining the concordance between bone age and chronological age despite his profound short stature. Our patient was diagnosed at 2 years and 9 months of age, significantly younger than most reported cases of SCKL5. Despite the progressive restriction in head growth, his current neurodevelopment and brain MRI remain normal. Nevertheless, given his young age, this atypical presentation of normal cognitive function must be interpreted with caution. Although his development is currently on track, he may still experience cognitive or DD later in life. Therefore, rather than prematurely classifying his condition as a definitively “mild” phenotype, we emphasize that his true neurodevelopmental trajectory has yet to be fully established. The current absence of obvious DD might result from variable penetrance or genetic modifiers; alternatively—and perhaps more likely—the patient is still in a preclinical stage before neurocognitive deficits become fully apparent. Thus, while his current developmental milestones are encouraging, rigorous longitudinal follow-up remains essential to accurately define his ultimate cognitive and developmental outcomes.

Molecular analysis identified two novel variants forming a compound-heterozygous CEP152 genotype (c.3661delA, p.(I1221Lfs*20), and c.1577+3A>G) in our proband, which were initially classified as “likely pathogenic” and “uncertain significance (VUS)”, respectively, based on ACMG/AMP guidelines. The c.3661delA variant truncates CEP152 within its C-terminal half (amino acids 804-1654) [27]. This C-terminal region is essential for centrosome biogenesis by mediating the interaction with CEP63 and maintaining proper centrosomal localization, both required for centriole duplication and maturation [27]. Truncation at p.I1221 disrupts this functional segment, likely impairing CEP63 binding and centrosome targeting to cause loss of function, consistent with the SCKL5 disease mechanism driven by biallelic loss-of-function CEP152 variants. We performed a minigene assay in 293T and HeLa cells to evaluate the splicing effect of c.1577+3A>G. RT-PCR densitometry revealed only ~30% canonical splicing product and ~70% aberrant transcripts (intron 12 retention and exon 12 skipping) in mutant-transfected cells. Both abnormal transcripts generate PTCs and are computationally predicted to yield truncated proteins with 550 and 479 amino acids. Degradation via NMD is only a bioinformatically inferred outcome and has not been experimentally validated [28,29]. Although our minigene assay revealed splice-disrupting effects in vitro, c.1577+3A>G remains formally classified as a VUS. These in vitro functional data provide supporting evidence for a splice-altering role of c.1577+3A>G but cannot definitively prove that identical splicing defects occur at the endogenous CEP152 locus in the patient. This offers a potential clue for interpreting the atypical clinical presentation of our proband. Because ~30% of transcripts retain normal splicing, a small amount of functional CEP152 protein could theoretically be produced. Residual normal protein from splice-disrupting variants has been hypothesized as one modulator of phenotypic heterogeneity in autosomal-recessive neurodevelopmental disorders [30]. Nevertheless, quantitative comparison of splicing results across studies is complicated by variable experimental systems, and minigene findings may not fully represent transcript profiles in disease-relevant neuronal tissue. Even so, these findings provide useful information for genetic counseling regarding future pregnancy risk for this couple.

This study has three main limitations. First, splicing alterations were validated exclusively in minigene-transfected cell lines instead of patient-derived primary cells or patient endogenous RNA. Our minigene construct only contains a partial fragment of intron 12; therefore, we cannot rule out the theoretical existence of distal intronic splicing-regulatory elements missing from this plasmid. As an inherent limitation of minigene systems, this artificial minigene construct may not fully recapitulate all regulatory features and physiological splicing patterns of the full-length endogenous CEP152 locus in vivo. Second, NMD-dependent degradation of mutant transcripts was merely bioinformatically predicted without quantitative detection of endogenous mutant RNA and truncated protein expression. Third, the gel-based minigene RT-PCR assay was conducted without biological replicates. Although Sanger sequencing confirmed the identities of splicing products, these in vitro splicing observations should be interpreted with caution.

## 5. Conclusions

We identified two novel compound heterozygous CEP152 variants, c.3661delA and c.1577+3A>G, in a patient with SCKL5. The in vitro minigene assay confirmed that the c.1577+3A>G splice variant perturbs canonical mRNA splicing. Our findings expand the spectrum of CEP152 variants associated with the patient’s phenotype and provide functional evidence for the splice-altering effect of c.1577+3A>G.

## Figures and Tables

**Table 1:** Clinical features and CEP152 variants of SCKL5.

Patient	Age/Sex	Genotype (Inheritance)	Height	Head Circ.	Craniofacial (SF, MG, FA, AE, BN)	Ocular (dPFs, Str, BP)	Oral/Dental (CP, HP, TA, ED)	Skeletal (11R, dBA, Sco, 5thC, BMP, PP)	Neuro/Genital (GA, ID, SG)	Reference
Patient 1 (Proband)	3 y 11 m/M	c.3661delA/ c.1577+3A>G (Comp. Het.)	Birth: −4.67 SD 3 y 11m: −4.38 SD	Birth: −3.33 SD 3 y 11m: −7.54 SD	SF, MG, AE, BN	dPFs, BP	HP	11R, Sco, 5thC		This study
Patient 442	10 y/M	c.261+1G>C (Hom.)	Birth: −5~−6 SD 10 y: −8~−9 SD	Birth: −3~−4 SD 10 y: −7~−8 SD	SF, MG, AE, BN	dPFs, Str, BP	HP, TA, ED	11R, dBA, 5thC, BMP, PP	GA (Crypt), ID, SG	Kalay 2011 [3]
Patient 443	8 y/F	c.261+1G>C (Hom.)	Birth: −6~−7 SD 8y: −4~−5 SD	Birth: −4~−5 SD 8 y: −7~−8 SD	SF, MG, AE, BN	dPFs, BP	HP, TA, ED	11R (11/12), 5thC, PP	GA (Clit), ID, SG	[3]
Patient 586	20 y/M	c.261+1G>C (Hom.)	20 y: −6~−7 SD	20 y: −10~−11 SD	SF, MG, AE, BN	dPFs	HP, TA, ED	11R, 5thC, PP	GA (Hypo), ID, SG	[3]
Patient 633	NA/F	c.261+1G>C (Hom.)	Birth: −7 SD 5.5 y: −7~−8 SD	Birth: −5~−6 SD	SF, MG, AE, BN	dPFs, Str, BP	CP, HP, TA, ED	11R (12/11), dBA, 5thC	GA (Clit), ID, SG	[3]
Patient 935	NA/M	c.261+1G>C (Hom.)	Birth: −6~−7SD 5 y: −5~−6 SD	Birth: −4 SD 5 y: −8 SD	SF, MG, AE, BN		HP, TA	11R (11/12), dBA, 5thC	ID, SG	[3]
Patient K1435	NA/F	c.2034T>G/ c.2694+1G>T (Comp. Het.)	Birth: −2.3 SD	Birth: −3.6 SD	SF, MG, AE, BN	dPFs	HP	dBA, 5thC (NA: 11R)	ID (NA: SG)	[3]
Patient AJ170	NA/M	c.261+1G>C (Hom.)	Birth: −5 SD 7 y: −5~−6 SD	Birth: −9 SD 3 y: −11 SD	SF, MG, FA, AE, BN	dPFs, Str	(NA: TA, ED)	NA	ID (NA: SG)	[3]
Patient AJ741	NA/M	c.2000A>G/ c.4210_4211delGT (Comp. Het.)	Birth: −3~−4 SD 13.5 y: −3~−4 SD	Birth: −7~−8 SD 13.5 y: −6~−7 SD	SF, MG, FA, BN		HP	Sco, 5thC (NA: dBA, PP)	ID (NA: SG)	[3]
Patient II-1	7 y/M	c.1060C>T/ c.1414-14A>G (Comp. Het.)	7 y: <−3 SD	7 y: <−3 SD	SF, MG, BN (NA: FA, AE)	BP (NA: dPFs, Str)	TA (NA: CP, HP)	5thC, PP (NA: 11R, dBA, Sco, BMP)	GA (Crypt), ID	[18]
Patient 11	7 y/M	c.1535T>A/ c.3346-5T>C (Comp. Het.)	7.3 y: −5.72 SD	7.3 y: <−3 SD	SF, MG, BN (NA: FA, AE)	NA	CP, HP, TA, ED	dBA (NA: Sco, BMP, PP)	ID	[19]
Patient 12	Prenatal (31 w)/M	c.3346-5T>C/ 129.6 kb del (Comp. Het.)	Birth/Fet.: <−3SD	24 w: <−3 SD 31 w: <−3 SD	NA	NA	NA	NA	NA	[20]
Patient 13	4 y 5 m/M	c.1917_1918insA/ c.3346-5T>C (Comp. Het.)	Birth: −4.67 SD 4 y 5m: −6.8 SD	4 y 5m: −10.3 SD	SF, MG, AE, BN	dPFs, BP	(NA: HP, TA, ED)	dBA, 5thC, BMP (NA: 11R, PP)	GA (Crypt), ID (NA: SG)	[21]
Frequency n/N (%)	10 M/3 F	-			SF: 12/12 (100%) MG: 12/12 (100%) FA: 2/10 (20%) AE: 9/10 (90%) BN: 12/12 (100%)	dPFs: 8/10 (80%) Str: 3/10 (30%) BP: 6/11 (55%)	CP: 2/11 (18%) HP: 9/10 (90%) TA: 7/9 (78%) ED: 5/9 (56%)	11R: 6/8 (75%) dBA: 6/8 (75%) Sco: 2/8 (25%) 5thC: 10/11 (91%) BMP: 2/9 (22%) PP: 4/8 (50%)	GA: 6/11 (55%) ID: 11/12 (92%) SG: 5/8 (63%)	

Abbreviations and Notes: Values list only positive clinical findings for brevity. Omitted traits are negative; missing data are denoted as [NA: …]. General: M, male; F, female; y, years; m, months; w, weeks; Fet., fetal; n, number of patients with the specific feature; N, total number of evaluated patients. Genotype: Hom., homozygous; Comp. Het., compound heterozygous. Growth Parameters: Head circ., head circumference; SD, standard deviation. Craniofacial: SF, sloping forehead; MG, micrognathia; FA, facial asymmetry; AE, low/malformed ears; BN, beaked nose. Ocular: dPFs, down-slanting palpebral fissures; Str, strabismus; BP, blepharophimosis. Oral/Dental: CP, cleft palate; HP, high-arched palate; TA, selective tooth agenesis; ED, enamel hypoplasia/defects. Skeletal: 11R, 11 pairs of ribs (11/12 denotes left/right rib count); dBA, delayed bone age; Sco, scoliosis; 5thC, 5th-finger clinodactyly; BMP, brachymesophalangia; PP, pes planus. Neuro/Genital: GA, genital anomalies [Crypt, cryptorchidism; Clit, clitoromegaly; Hypo, hypospadia]; ID, intellectual disability; SG, simplified gyri. Published clinical data of Patient II-1, Patient 11, Patient 12 and Patient 13 were obtained from previously reported case studies [18], [19], [20] and [21], respectively.

## Data Availability

The variant data generated in this study were deposited in ClinVar (NCBI) under accession numbers SCV006324105 and SCV006324106 (https://www.ncbi.nlm.nih.gov/clinvar/, accessed on 7 October 2026).

## Supplementary Materials

The following supporting information can be downloaded at: https://www.mdpi.com/article/10.3390/genes17101249/s1, Figure S1: Full uncropped original agarose gel image corresponding to Figure 4B; Table S1: Variant classification assessment according to ACMG/AMP 2015 and ClinGen SVI recommendations; Table S2: Raw densitometry data underlying Figure 4E.
